# Extracellular vesicle proteome unveils cathepsin B connection to Alzheimer’s disease pathogenesis

**DOI:** 10.1093/brain/awad361

**Published:** 2023-12-10

**Authors:** Kohei Yuyama, Hui Sun, Risa Fujii, Isao Hemmi, Koji Ueda, Yukifusa Igeta

**Affiliations:** Lipid Biofunction Section, Faculty of Advanced Life Science, Hokkaido University, Sapporo 001-0021, Japan; Lipid Biofunction Section, Faculty of Advanced Life Science, Hokkaido University, Sapporo 001-0021, Japan; Cancer Proteomics Group, Cancer Precision Medicine Center, Japanese Foundation for Cancer Research, Tokyo 035-8550, Japan; Department of Nursing, Japanese Red Cross College of Nursing, Tokyo 150-0012, Japan; Cancer Proteomics Group, Cancer Precision Medicine Center, Japanese Foundation for Cancer Research, Tokyo 035-8550, Japan; Department of Dementia, Dementia Center, Toranomon Hospital, Tokyo 105-8470, Japan; Division of Dementia Research, Okinaka Memorial Institute for Medical Research, Tokyo 105-8470, Japan

**Keywords:** extracellular vesicle, Alzheimer’s disease, ATN classification, cathepsin B, blood biomarker

## Abstract

Extracellular vesicles (EVs) are membrane vesicles that are released extracellularly and considered to be implicated in the pathogenesis of neurodegenerative diseases including Alzheimer’s disease. Here, CSF EVs of 16 ATN-classified cases were subjected to quantitative proteome analysis. In these CSF EVs, levels of 11 proteins were significantly altered during the ATN stage transitions (*P* < 0.05 and fold-change > 2.0). These proteins were thought to be associated with Alzheimer’s disease pathogenesis and represent candidate biomarkers for pathogenic stage classification. Enzyme-linked immunosorbent assay analysis of CSF and plasma EVs revealed altered levels of cathepsin B (CatB) during the ATN transition (seven ATN groups in validation set, *n* = 136). The CSF and plasma EV CatB levels showed a negative correlation with CSF amyloid-β_42_ concentrations. This proteomic landscape of CSF EVs in ATN classifications can depict the molecular framework of Alzheimer’s disease progression, and CatB may be considered a promising candidate biomarker and therapeutic target in Alzheimer’s disease amyloid pathology.

## Teaser

Yuyama *et al.* analyse the proteome of extracellular vesicles isolated from the CSF of patients with Alzheimer’s disease, and find changes in the levels of a number of protein species, particularly cathepsin B, in association with disease progression. Cathepsin B may be a promising candidate for an Alzheimer’s disease diagnostic biomarker.

## Introduction

Alzheimer’s disease (AD) is the most common cause of dementia and a major medical problem to be solved worldwide. The disease is pathologically characterized by amyloid plaques composed of extracellular aggregations of amyloid-β peptide (Aβ) and intracellular deposits of neurofibrillary tangles composed of phosphorylated Tau protein (pTau).^1,2^ Aβ_42_ in the CSF declines about 25 years before AD onset, and CSF pTau increases 10 years before.^1^ The amyloid cascade hypothesis^3^ suggests that Aβ deposition triggers a sequence of subsequent neurotoxicity (due to pTau) and neuroinflammation (due to activated microglia). The pathological classification of AD progression at each clinical stage is known as Braak staging; CSF levels of Aβ_42_ and pTau reflect the histopathological characteristics and Braak stage of the disease.^4,5^ In recent years, the spread of pathogenic proteins between neurons by extracellular vesicles (EVs) has been suggested to be involved in AD progression.^6^

EVs such as exosomes (50–150 nm), microvesicles (150–1000 nm) and apoptotic vesicles (1000–5000 nm) are secretory membrane capsules that contain multiple proteins, lipids and nucleic acids. The nature and quantity of their cargoes depend on the cell type and often on the pathological state of the donor cell.^7,8^ EVs are released from a variety of cells, including those in the CNS,^9^ and it has been reported that those derived from the CNS contain molecules such as Aβ, its catabolic enzymes, and tau, which all play important roles in the pathogenesis of AD.^6,10,11^ Previously, we reported that neuron-derived EVs trap and transfer Aβ to microglia for degradation.^12,13^ Moreover, inhibition of EV production has been found to decrease amyloid deposition in a mouse model of AD.^14^ Furthermore, EVs can mediate the neuron–neuron spread of pathogenic tau species.^15–17^ Since EVs can be collected from various body fluids, including blood, urine and CSF, the molecules they carry can be considered as circulating surrogates, from which molecular information about their donor cells can be accessed in a minimally invasive manner (i.e. blood-based), allowing the long-term continuous monitoring of disease status.

Recently, trends towards the early diagnosis of AD have been increasing along with the development of disease-modifying drugs. In 2011, the National Institute on Aging and the Alzheimer’s Association (NIA-AA) classified the entire course of AD into: (i) preclinical stage; (ii) mild cognitive impairment (MCI) due to AD; and (iii) AD dementia. Preclinical diagnosis of AD is possible if CSF Aβ_42_ levels decline.^18^ In 2018, the NIA-AA developed guidelines to group the pathological processes of AD according to Aβ deposition (A), pathological tau (T) and neurodegeneration (N) (ATN) biomarkers, resulting in a transition from a clinical to biochemical diagnosis. ATN classification was initially developed for research purposes; however, it has gradually been applied to clinical medicine. This system ultimately enables early (preclinical phase) diagnosis based on biomarkers.

In this study, using ATN-classified samples, novel pathogenic factors and biomarkers were investigated by targeting EVs. We collected and stored CSF and plasma samples using carefully unified collection procedures and biobanking protocols and performed ATN classification. We excluded groups showing non-AD pathologic changes, instead choosing patients with normal AD biomarkers and those on the AD continuum. We then performed proteomic profiling of CSF EVs according to the ATN classification to identify unique proteins that exhibited level changes in line with the pathogenic progression of the disease. The acquired proteins were shown not only to be involved in AD pathogenesis but also highlighted as potential biomarkers and therapeutic targets.

## Materials and methods

### Patients

Eligible participants were 136 patients (male: *n* = 71, female: *n* = 65, age: 71.3 ± 7.4 years) who visited the outpatient clinic of the Dementia Department of Toranomon Hospital from April 2017 to April 2021 and gave consent for lumbar puncture to collect CSF. All participants were Japanese. A detailed current medical history and medical interview were obtained, and physical and neurological examinations were performed. Neuropsychological tests included the Mini Mental State Examination and the Alzheimer’s Disease Assessment Scale-cognitive subscale (Japanese version). The Weschler Memory Scale-Revised was performed as an aid in the diagnosis of MCI. The Geriatric Depression Scale-15 was used to exclude depression.

Imaging studies included MRI and ^123^I-*N*-isopropyl-p-iodoamphetamine (IMP) brain perfusion single-photon emission computed tomography (SPECT). Conditions such as dementia, several neurodegenerative diseases, trauma, psychiatric disorders and medical conditions were excluded as follows: vascular dementia, frontotemporal lobar dementia, progressive supranuclear palsy, corticobasal degeneration, multiple system atrophy, head trauma, epileptic seizures, alcoholism, depression, normal pressure hydrocephalus, hepatic encephalopathy and hypothyroidism. Lewy body dementia was excluded with the help of ^123^I-metaiodobenzylguanidine (MIBG) and dopamine transporter scanning. Subjects included 50 patients with AD (male: *n* = 22, female: *n* = 28, age: 71.9 ± 7.6 years), 26 patients with MCI (male: *n* = 14, female: *n* = 12, age: 72.5 ± 7.1 years) and 60 normal control participants (male: *n* = 35, female: *n* = 25, age: 70.3 ± 7.3 years). AD was diagnosed using the Text Revision of the Diagnostic and Statistical Manual of Mental Disorders-IV (DSM-IV-TR) and as clinically defined probable AD according to the National Institute of Neurological Communicative Disorders and Stroke–Alzheimer’s Disease and Related Disorders Association (NINCDS-ADRDA) criteria.^19^ MCI was diagnosed according to the clinical criteria of the Association Workgroup of the National Institute on Aging Alzheimer’s Disease^20^ and classified as amnesic type. All the neuropsychological tests and imaging diagnoses mentioned above were used as clinical aids by experienced neurologists and geriatricians who strictly adhered to the diagnostic criteria and made precise diagnoses.

This study was conducted in accordance with the ethical guidelines for medical and health research involving human subjects in Japan and in compliance with the Declaration of Helsinki. The study protocol was approved by the Clinical Research Ethics Committee of Toranomon Hospital. Detailed information was provided to patients and their families, and all participants provided written informed consent.

### Collection of CSF and plasma samples

CSF and blood samples were collected at the Department of Dementia, Toranomon Hospital. Participants indicated for lumbar puncture were 41–80 years of age, did not take antiplatelet or anticoagulant drugs and had no blood disorders. After overnight fasting, lumbar puncture was performed between 9:00 a.m. and 10:00 a.m. The spinal needle used for CSF was a 23–25 G top spinal needle with a stainless steel and polypropylene needle base (23–25 G × 70 mm, Hakko). CSF was collected in drops in sterile 10 ml polypropylene spit tubes (Asiakizai). This process was always performed by one experienced physician using the same technique and equipment at the same time of day. The collected CSF samples were centrifuged at 3500 rpm (2200*g*) at room temperature for 7 min to sediment cells and other insoluble material. The supernatant was then dispensed into polypropylene microtubes (1.5 ml, Thermo Fisher Scientific) at 500 µl each with a polypropylene dispensing tip for enzyme-linked immunosorbent assay (ELISA). For EV analysis, 500 µl of the CSF was dripped directly from a spinal needle into a polypropylene microtube, and specimens contaminated with blood were discarded.

Immediately after CSF collection, whole blood was collected from the left upper arm into a vacuum tube containing EDTA2Na (Sekisui Medical). Plasma was separated by centrifugation at 2200*g* and 4°C for 15 min and dispensed in approximately 500 μl portions into the same type of container as the CSF. All samples were frozen at 80°C within 1 h after collection and banked until the analysis. Only one freeze-thaw cycle was performed.

### ATN classification

Subjects were classified according to their ATN profile, using CSF Aβ_42_ for amyloid deposition (A), CSF pTau for pathologic tau (T) and CSF tTau for neurodegeneration (N). CSF biomarkers were measured using the following ELISAs: a human Aβ_1–42_ ELISA (Fujifilm Wako), finoscolor pTau kit with Tau mAb T270 (10–994, Nipro) and Tau (Total) Human ELISA Kit (ThermoFisher). First, a receiver operating characteristic (ROC) analysis that separated non-AD (control and MCI) from AD was performed on a subset of the cohort (*n* = 60) to determine the cut-off levels for each biomarker. Cut-off values were determined by selecting the point on the ROC curve closest to the top-left corner using R, version 4.1.1 (Supplementary Fig. 1 and Supplementary Table 2). To select the discovery cohort representing precise pathological profiles of AD, samples close to the cut-off threshold were omitted, and those exhibiting typical clinical symptoms were included. The discovery cohort consisted of four controls (A−T−N−), eight cases with amyloid deposition but no tau pathology (four A+T−N− and four A+T−N+) and four cases with both amyloid and tau pathology (A+T+N+). Cases without amyloid pathology but with neurodegeneration and tau pathology were referred to as non-AD pathologic change and excluded from the proteomics analysis to increase the likelihood of identifying proteins related to AD. Finally, the entire cohort (*n* = 136) was classified according to the ATN profiling and used to validate the proteomics findings.

### Mass spectrometric analysis of CSF extracellular vesicles

CSF samples (500 μl) were centrifuged at 2000*g* for 15 min, and then EVs were purified from the supernatants using EVSecond L70 columns (GL Sciences Inc.) according to the manufacturer’s instructions. The size of the isolated EVs was measured with an interferometric light microscopy (ILM)-based nanoparticle analyser (Videodrop; Myriade); at least 300 particles were counted for each measurement. The EV fractions were dried and resolved in 30 μl of 1 × Laemmli’s sample buffer. After reduction with 10 mM Tris (2-carboxyethyl) phosphine (TCEP) at 100°C for 10 min and alkylation with 50 mM iodoacetamide at ambient temperature for 45 min, protein samples were subjected to SDS-PAGE. The electrophoresis was stopped at the migration distance of 2 mm from the top edge of the separation gel. After Coomassie Brilliant Blue (CBB)-staining, protein bands were excised, destained and cut finely prior to in-gel digestion with Trypsin/Lys-C Mix (Promega) at 37°C for 12 h.

The resulting peptides were extracted from gel fragments and analysed with an Orbitrap Fusion Lumos mass spectrometer (Thermo Scientific) combined with an UltiMate 3000 RSLC nano-flow high-performance liquid chromatography system (Thermo Scientific). Peptides were enriched with a μ-precolumn (0.3 mm i.d. × 5 mm, 5 μm, Thermo Scientific) and separated on AURORA columns (0.075 mm i.d. × 250 mm, 1.6 μm, Ion Opticks Pty Ltd.) using a two-step gradient: 2–40% acetonitrile for 110 min, followed by 40–95% acetonitrile for 5 min in the presence of 0.1% formic acid. The analytical parameters of the Orbitrap Fusion Lumos were set as follows: resolution of full scans = 50,000; scan range (m/z) = 350–1500; maximum injection time of full scans = 50 ms; AGC target of full scans = 4 × 10^5^; dynamic exclusion duration = 30 s; cycle time of data dependent tandem MS (MS/MS) acquisition = 2 s; activation type = HCD; MS/MS detector = ion trap; maximum injection time for MS/MS = 35 ms; AGC target of MS/MS = 1 × 10^4^.

The MS/MS spectra were searched against the *Homo sapiens* protein sequence database in SwissProt using Proteome Discoverer 2.4 software (Thermo Scientific), in which peptide identification filters were set at a false discovery rate < 1%. Label-free relative quantification analysis of proteins was performed with the default parameters of Minora Feature Detector node, Feature Mapper node and Precursor Ions Quantifier node in Proteome Discoverer 2.4 software. The LC/MS raw data files were deposited in PXD043418 for ProteomeXchange and JPST002225 for jPOST.

### Immunoassay of CSF and plasma extracellular vesicles

EVs were collected from CSF and plasma by differential ultracentrifugation,^21^as different isolation methods were tested to determine if they produced equivalent results. CSF (100 µl) and plasma (400 µl) were sequentially centrifuged at 2000*g* for 10 min and 10 000*g* for 30 min at 4°C, to remove cells and debris, and then again at 100 000*g* for 1 h at 4°C to pellet the EVs. The EVs were lysed in 6 µl of 0.1% SDS/PBS, mixed with 0.66 µl of 10% Triton X-100/PBS and applied to ELISA plate wells. The levels of cathepsin B (CatB) were determined using sandwich ELISA (CatB: Abcam #ab119584).

### Statistical analysis

ROC analysis was performed in R (version 4.1.1). All other statistical analyses and graph preparations were performed using GraphPad Prism 9 software (San Diego, CA, USA). Data are expressed as mean ± SEM. Unpaired Student’s *t*-tests were used to compare two groups and one-way ANOVA followed by Tukey’s multiple comparisons tests were used to compare more than two groups. Correlation analysis was performed using Pearson’s test. Statistical significance was set at *P* < 0.05; *P < 0.05, **P < 0.01, ***P < 0.001. All other experiment information, including sample size, can be found in the figure legends.

## Results

### Proteomic analysis of CSF extracellular vesicles in the ATN classification

**Figure 1:**
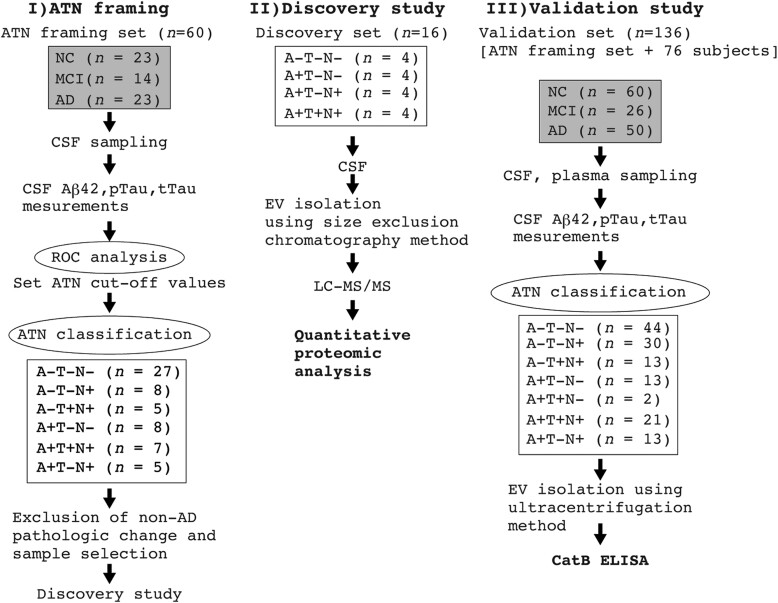
**Workflow of the analysis of extracellular vesicle (EV) proteins in CSF and plasma according to ATN classification**. CSF and plasma samples were collected from normal control (NC), mild cognitive impairment (MCI), and Alzheimer’s disease (AD) participants and classified into ATN groups based on the levels of Aβ_42_, pTau and tTau in the CSF. In the liquid chromatography-mass spectrometry-based biomarker discovery phase, after the exclusion of non-AD pathologic changes, EVs were isolated from the 16 selected CSF samples using size exclusion chromatography and analysed by mass spectrometry. In an enzyme-linked immunosorbent assay (ELISA)-based validation study for CatB, EVs were collected from 136 CSF and plasma samples by ultracentrifugation prior to the ELISA. LC-MS/MS= liquid chromatography-tandem mass spectrometry; ROC = receiver operating characteristic.

The workflow is shown in Fig. 1. First, cut-off values for CSF Aβ_42_, pTau and tTau were calculated using ROC analysis on a subset of the data (*n* = 60; Supplementary Table 1). The resulting cut-off values were 677.9 pg/ml (AUC 0.80, sensitivity 87%, specificity 70%) for Aβ_42_, 73.0 pg/ml (AUC 0.74, sensitivity 97%, specificity 57%) for pTau and 597.7 pg/ml (AUC 0.80, sensitivity 78%, specificity 70%) for tTau (Supplementary Fig. 1 and Supplementary Table 2). Next, these cut-off values were used to design a discovery set for proteomics analysis (Table 1).

**Table 1:** Patient information: discovery set (for proteomic analysis)

Classification	A−T−N−	A+T−N−/A+T−N+	A+T+N+
*n*	4	8 (4/4)	4
Age, mean ± SD	75.5 ± 4.4	73.4 ± 5.7	65.3 ± 11.0
Sex	4 F	2 M, 6 F	2 M, 2 F
Diagnosis	4 NL	1 NL, 3 MCI, 4 AD	4 AD
MMSE	28.75± 1.5	26.8 ± 2.9	23.3 ± 4.3
Aβ_1–42_ (pg/ml)	1326.8 ± 316.0	434.7 ± 70.8	324.8 ± 109.3
pTau (pg/ml)	42.0 ± 6.5	60.0 ± 16.8	129.3 ± 35.9
tTau (pg/ml)	341.7 ± 81.9	579.6 ± 187.8	1355.0 ± 445.0

A = Aβ deposition; Aβ = amyloid beta; AD = Alzheimer’s disease; F = female; M = male; MCI =mild cognitive impairment; MMSE = Mini Mental State Examination; N = neurodegeneration; NL = normal; T = pathological tau.

**Figure 2:**
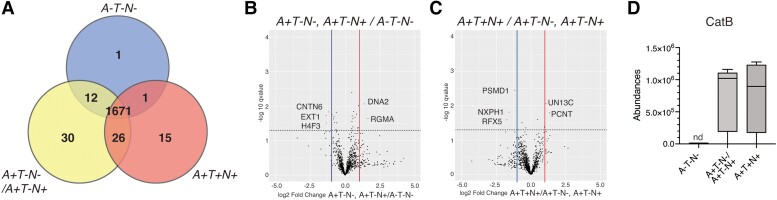
**Proteomic analysis of CSF extracellular vesicle (EV) proteins in the discovery set under ATN classification**. (**A**) Venn diagram of CSF EV proteins quantified in three ATN groups. (**B** and **C**) Volcano plots showing statistically significant EV proteins in A+T−N−/A+T−N+ versus A−T−N− (**B**) and in A+T+N+ versus A+T−N−/A+T−N+ (**C**). (**D**) The abundances of CatB protein quantified by mass spectrometry are shown. A = Aβ deposition; N = neurodegeneration; nd = not detected; T = pathological tau.

To identify proteins associated with the progression of AD pathology, EVs were isolated from the CSF of 16 participants (discovery set), the particle size was measured and particles were analysed using LC-MS/MS. The isolated EVs had an average particle size of around 120 nm (Supplementary Fig. 2). In total, 1756 proteins were identified and quantified from the CSF EVs (Supplementary Table 3). The identified CSF EV proteins were compared with the EV proteins in the top100 list of the Vesiclepedia database^22,23^ (Supplementary Fig. 3A) and 87 proteins from the top100 list were found in the CSF EV proteomics list. The CSF EV proteins were further functionally evaluated by gene ontology (GO) analysis with the Database for Annotation, Visualization and Integrated Discovery (DAVID).^24^ The enriched proteins in biological process, molecular function and cellular component were predominantly associated with extracellular structural constituent and extracellular structure organization (Supplementary Fig. 3B). These results suggested that the samples were enriched in EVs. A total of 1487, 1592 and 1535 proteins were identified in the CSF EVs of the A−T−N−, A+T-N−/A+T−N+ and A+T+N+ groups, respectively. Approximately 95.2% of the proteins (1671 proteins) were detected in all groups, with 1, 30 and 15 proteins unique to A−T−N−, A+T−N−/A+T−N+ and A+T+N+, respectively (Fig. 2A and Supplementary Table 4). The numbers of proteins shared by the two groups were: 12 (A−T−N− and A+T−N−/A+T−N+), 26 (A+T−N−/A+T−N+ and A+T+N+) and 1 (A−T−N− and A+T+N+) (Supplementary Table 5). The abundance levels of the EV marker proteins Alix, CD9 and CD63 did not differ between the three groups (Supplementary Fig. 4), indicating that variances in proteomic profiles are probably not due to differences in EV number/composition.

Figure 2B and C shows volcano plots of 1485 and 1519 common proteins detected between the A−T−N− and A+T−N−/A+T−N+ groups and between the A+T−N− and A+T+N+ groups, respectively. A total of 11 EV proteins varied significantly among the three ATN groups. In the transition from A−T−N− to A+T−N−/A+T−N+, three proteins (repulsive guidance molecule A, DNA replication ATP-dependent helicase/nuclease DNA2 and CatB) were significantly upregulated, while another three proteins (contactin 6, extosin-1 and histone H4) were significantly downregulated (as determined by *P* < 0.05 and |log2 fold change| > 1; Fig. 2B, Table 2 and Supplementary Figs 5A and 6). On the other hand, in the transition from A+T−N−/A+T−N+ to A+T+N+, two proteins (pericentrin and protein unc-13 homolog C) were significantly upregulated, whereas three proteins (26S proteasome non-ATPase regulatory subunit 1, neurexophilin-1 and DNA-binding protein RFX5) were significantly downregulated (as determined by *P* < 0.05 and |log2 fold change| > 1; Fig. 2C, Table 2 and Supplementary Fig. 5B). In addition, the levels of proteins such as CatB, contactin 6, extosin 1, protocadherin 1 and pericentrin were significantly altered between A−T−N− and A+T+N+ (Supplementary Table 6). The transition from A−T−N− to A+T−N−/A+T−N+ corresponds with Aβ pathogenesis, and the transition from A+T−N− to A+T+N+ corresponds with tau pathogenesis.^19^ Thus, we successfully identified several unique proteins as potential biomarkers to distinguish pathological stages of AD, possibly reflecting their involvement in brain cell pathological changes.

**Table 2:** Up- and down-regulated EV proteins during ATN stage transition

Protein ID	Protein name	log2FC	q-value
**A+T−N−, A+T−N+/A−T−N−**			
Q96B86	Repulsive guidance molecule A (RGMA)	1.64	2.3 × 10^−2^
P51530	DNA replication ATP-dependent helicase/nuclease DNA2 (DNA2)	1.35	8.6 × 10^−3^
Q9UQ52	Contactin 6 (CNTN6)	−1.21	1.5 × 10^−2^
P62805	Histone H4 (H4F3)	−1.36	3.1 × 10^−2^
Q16394	Exostosin-1 (EXT1)	−1.37	1.9 × 10^−2^
P07858	Cathepsin B (CatB)	Detected only in A+ group	
**A+T+N+/A+T−N−, A+T−N+**			
O95613	Pericentrin (PCNT)	1.32	1.6 × 10^−2^
Q8NB66	Protein unc-13 homolog C (UN13C)	1.08	8.7 × 10^−3^
Q99460	26S proteasome non-ATPase regulatory subunit 1 (PSMD1)	−1.19	3.6 × 10^−3^
P58417	Neurexophilin-1 (NXPH1)	−1.59	1.5 × 10^−2^
P48382	DNA-binding protein RFX5 (RFX5)	−1.75	2.6 × 10^−2^

### Elevated CatB levels in CSF and plasma extracellular vesicles during amyloid pathogenesis

**Figure 3:**
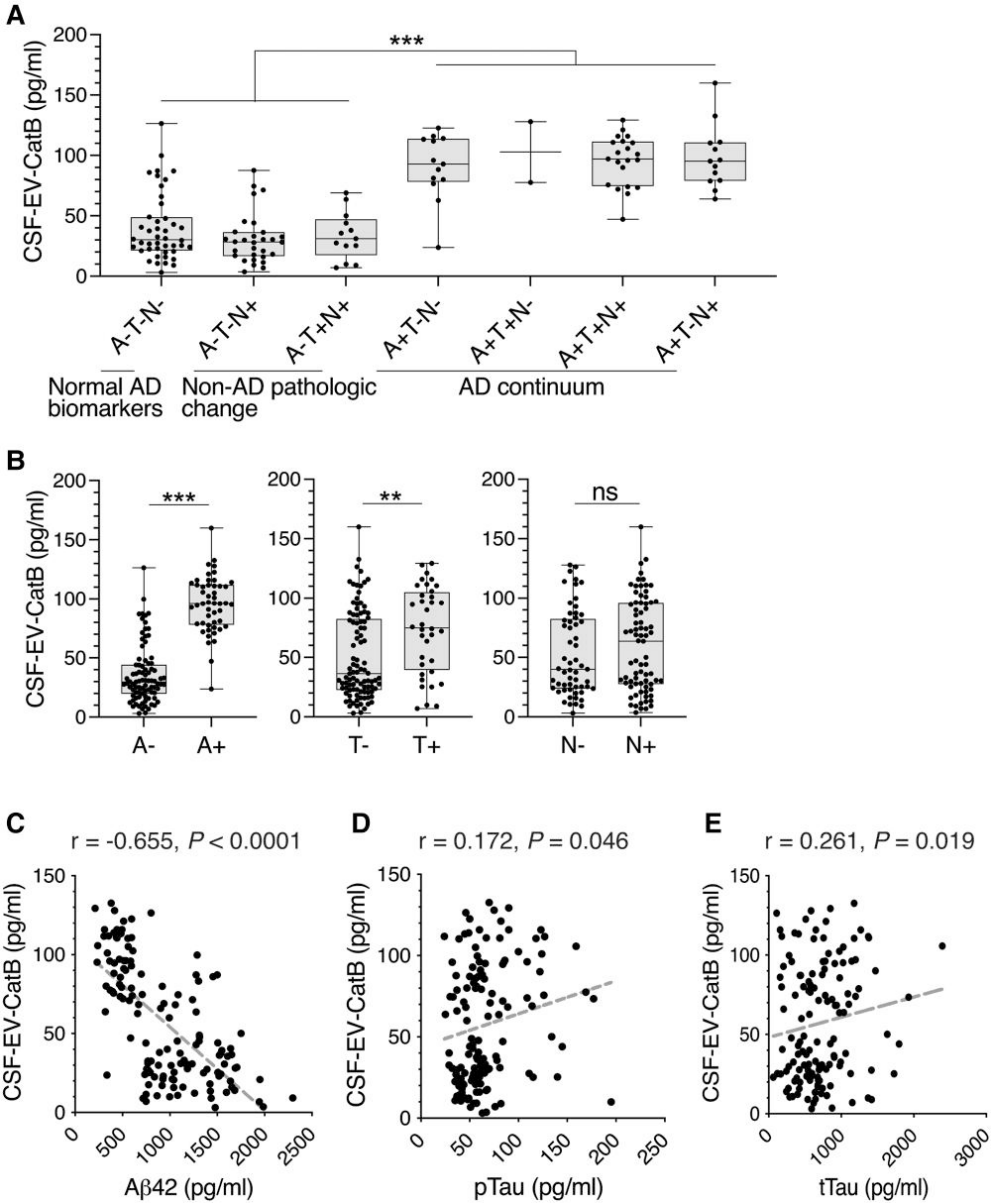
**ELISA-based validation of CatB in CSF extracellular vesicles (EVs).** (**A**) Box plots of CatB abundances in CSF EVs in each ATN group, which were quantified by ELISA (validation set, *n* = 136). (**B**) Box plots of CatB in CSF EVs in A+/A−, T+/T and N+/N− classifications. One-way ANOVA, followed by Tukey’s multiple comparisons test. ns = non-significant, **P < 0.01, ***P < 0.005. (**C**–**E**) Scatter plots showing correlations between CatB in CSF EVs and (**C**) Aβ_42_, (**D**) pTau and (**E**) total Tau levels in CSF. AD = Alzheimer’s disease; Aβ = amyloid beta; A = Aβ deposition; N = neurodegeneration; T = pathological tau.

CatB, one of the proteins that significantly changes between the A−T−N− and A+T−N−/A+T−N+, is associated with senile plaques in AD brains and is also involved in the lysosomal protease-mediated processing of amyloid precursor protein (APP) to Aβ.^25,26^ Therefore, we have selected CatB for the validation of proteomics findings in a larger cohort using both CSF and plasma EVs. EVs were isolated from 136 CSF samples, categorized into seven ATN groups (validation set, Table 3), and their CatB levels (CSF-EV-CatB) were determined by ELISA. The EVs isolated from both CSF and plasma had an average particle size of around 105 nm (Supplementary Fig. 2). Consistent with the proteomic analysis, CSF-EV-CatB levels were significantly increased (A−, 35.9 ± 24.7 pg/ml; A+, 95.4 ± 23.9 pg/ml; *P* < 0.0001) in the amyloid positive groups compared with the amyloid negative groups (Fig. 3A), and the levels at A+ and T+ were significantly higher than those at A− and T−, respectively (Fig. 3B). CatB concentrations showed a strong negative correlation with CSF Aβ_42_ concentrations (r = −065, *P* < 0.0001), a very weak correlation (r = 0.172, *P* = 0.046) with pTau and a weak correlation with tTau (*r* = 0.261, *P* = 0.019) (Fig. 3C–E). These results indicated that CSF-EV-CatB is strongly associated with the Aβ status of CSF.

**Table 3:** Patient information: validation set for CatB enzyme-linked immunosorbent assay

Classification	Normal AD biomarkers	Non-AD pathologic change			AD continuum		
	A−T−N−	A−T−N+	A−T+N+	A+T−N−	A+T+N−	A+T+N+	A+T−N+
*n*	44	30	13	13	2	21	13
Age, mean ± SD	70.2 ± 8.4	72.5 ± 6.1	74.8 ± 4.1	69.1 ± 7.6	74.0 ± 4.2	69.8 ± 8.0	73.2 ± 7.5
Sex	28 M, 16 F	14 M, 16 F	8 M, 5 F	5 M, 8 F	2 F	10 M, 11 F	6 M, 7 F
Diagnosis	31 NL, 10 MCI, 3 AD	20 NL, 6 MCI, 4 AD	4 NL, 1 MCI, 8 AD	1 NL, 1 MCI, 11 AD	2 MCI	1 NL, 2 MCI, 18 AD	3 NL, 4 MCI, 6 AD
MMSE	28.6 ± 1.5	27.9 ± 2.7	23.2 ± 6.4	22.9 ± 3.1	26.5 ± 2.1	24.3 ± 3.7	25.0 ± 4.3
Aβ_1–42_ (pg/ml)	1184.0 ± 291.6	1316.8 ± 417.9	1063.1 ± 361.8	453.1 ± 108.1	392.2 ± 33.5	465.5 ± 128.4	464.5 ± 107.8
pTau (pg/ml)	47.5 ± 10.0	55.1 ± 11.0	109.8 ± 36.2	52.3 ± 13.7	122.0 ± 66.5	107.1 ± 26.5	54.2 ± 16.7
tTau (pg/ml)	379.3 ± 149.0	755.8 ± 105.5	1271.0 ± 315.1	418.6 ± 161.3	566.0 ± 24.5	1116.5 ± 417.1	939.4 ± 237.8

A = Aβ deposition; Aβ = amyloid beta; AD = Alzheimer’s disease; F = female; M = male; MCI = mild cognitive impairment; MMSE = Mini Mental State Examination; N = neurodegeneration; NL = normal; T = pathological tau.

**Figure 4:**
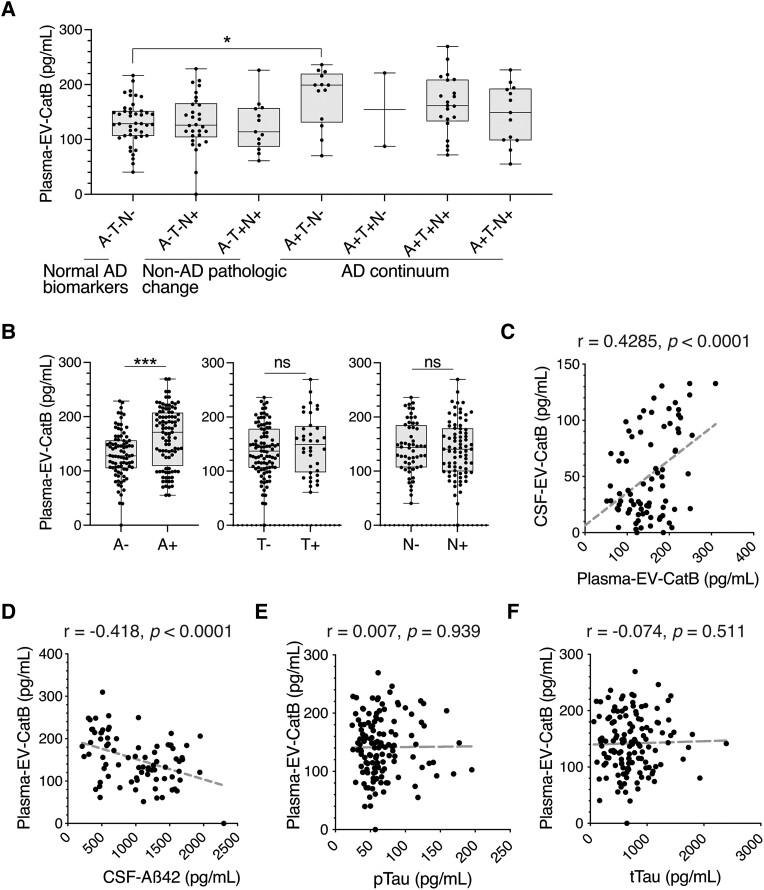
**ELISA validation of CatB in plasma extracellular vesicles (EVs) as a putative blood biomarker using ATN classification**. (**A**) Box plots of CatB abundances in plasma EVs in each ATN group, which were quantified by ELISA (validation set, *n* = 136). (**B**) Box plots of plasma-EV-CatB in A+/A−, T+/T− and N+/N− classifications. One-way ANOVA, followed by Tukey’s multiple comparisons test. ns = non-significant, *P < 0.05 and ***P < 0.005. (**C**) Correlations between CatB levels in EVs derived from CSF and plasma. (**D**–**F**) Scatter plots showing correlations between plasma-EV-CatB and (**D**) Aβ_42_, (**E**) pTau and (**F**) total Tau levels in CSF. A = Aβ deposition; AD = Alzheimer’s disease; N = neurodegeneration; T = pathological tau.

EVs in peripheral blood can be used to monitor pathological changes in the CNS.^27^ Therefore, we measured both total CatB and EV CatB using plasma samples from 136 subjects by ELISA (validation set, Table 3). As the result, the concentrations of plasma CatB did not significantly differ among the ATN groups (Supplementary Fig. 7A and B). In contrast to free plasma CatB, plasma-EV-CatB levels were significantly increased in A+T−N− compared with A−T−N− (A−T−N−, 143.3 ± 40.4 pg/ml; A+T−N−, 177.7 ± 52.6 pg/ml; *P* = 0.042) but not in the other AD continuum groups (Fig. 4A). The levels were significantly higher in A+ than A−, but not between T+/T− or N+/N− (Fig. 4B). Plasma-EV-CatB showed a moderate negative correlation with CSF Aβ_42_ concentrations but not with CSF pTau or tTau (Fig. 4C–F). These results suggested that plasma-EV-CatB is associated with CSF Aβ status.

## Discussion

In the present study, normal controls and subjects with a clinical diagnosis of AD or Mci were categorized according to the ATN classification as the following: (i) normal AD biomarker group; (ii) AD continuum group; and (iii) non-AD pathologic change group, based on CSF Aβ_42_, pTau and tTau concentrations. Quantitative proteomics analysis by MS identified 1756 proteins in CSF EVs, including 11 proteins with significant differences among the ATN groups. Of these, CatB was validated by ELISA with a larger number of CSF and plasma samples, confirming that EV CatB levels in CSF and plasma increased from the A− to A+ shift and correlated with CSF Aβ_42_. These results implied that EV CatB might be involved in amyloid pathology.

The ATN classification system used in this study is an unbiased system for classifying participants by pathological process.^28^ The cut-off value of Aβ_42_ in CSF, which is used as a valid indicator of A, associated with amyloidogenesis, varies among medical institutions, because Aβ_42_ is prone to degeneration according to the collection method and storage conditions, causing confusion in diagnosis and research.^29^ In this study, we therefore conducted CSF sampling using a consistent method at a single institution. This enabled us to achieve stable and accurate ATN classification and carry out a rigorous proteomic analysis.

The ELISA-based values of CSF Aβ_42_ in this study were used to frame the A group instead of PET imaging. Amyloid PET and CSF biomarkers can identify early AD with high accuracy.^30^ However, a recent study reported that the earliest Aβ changes are more likely to be observed with CSF Aβ measurements than with amyloid PET in the elderly.^31^ This suggests that the ATN classification with ELISA-based CSF Aβ values represents optimal classification for understanding pathophysiological AD progression in non-demented elderly individuals and for identifying ATN biomarker profiles. It is reported that approximately 30% of non-AD pathologic changes are included as part of the clinical diagnosis of AD.^32^ Narrowing down EVs associated with Aβ pathology by removing non-AD pathologic change samples may increase the success rate of precisely identifying AD biomarkers.

Recent studies have shown that analysis of EVs in peripheral blood provides access to proteomic and transcriptomic profiles of neurons and other brain cells in living human individuals.^33,34^ EVs and their luminal cargos are highly stable in storage and in body fluid circulation, because they are protected by a lipid bilayer.^35^ This property may allow certain EV proteins to be more useful as blood biomarkers than free proteins. In addition, although further studies are needed, if specific EV proteins are involved in amyloid and tau pathology, they may provide potential biomarkers that can be used for more accurate and earlier detection compared with Aβ and tau. Proteomic studies have identified several EV proteins that vary between healthy subjects and AD.^36,37^

Of the 11 EV proteins in this study, CatB has been implicated in several neurodegenerative diseases such as AD and traumatic brain injury.^38,39^ In AD brains, CatB levels are elevated in activated astrocytes around neuritic plaques.^26,38,40^ In the amyloid cascade hypothesis, Aβ is generated by the processing of APP by β-site APP cleaving enzyme 1 (BACE1) and γ-secretase.^41^ However, several reports have shown that CatB is involved in Aβ processing; in an *in vitro* study, CatB cleaved APP much faster than BACE1.^42^ In this report, CatB co-localized with APP and Aβ in regulated secretory vesicles and produced >90–95% of Aβ, whereas BACE1 produced < 5–10% of Aβ in the constitutive secretory pathway.^43,44^ Furthermore, CatB is produced by astrocytes and is believed to excise Aβ_2–x_ from APP, while BACE1 is believed to excise Aβ_1–x_ from APP. Using cultured cells and mouse AD models, pharmaceutical and genetic inhibition of CatB has been shown to reduce Aβ release and amyloid deposition and improve memory impairment.^25,44^ Recently, a strong correlation between severe periodontal disease and cognitive decline has been reported. Chronic systemic exposure to lipopolysaccharide from the periodontogenic bacteria *Porphyromonas gingivalis* increases CatB expression in neurons in middle-aged mice and induces AD-like phenotypes with Aβ accumulation in neurons.^45^ In this study, EV CatB levels in plasma correlated with those in CSF, and both correlated with CSF Aβ_42_ and were elevated in the A− to A+ shift. EV CatB levels in CSF also significantly varied between T− and T+ (Fig. 3B), which may be due to the different proportions of A-positive individuals in each group (T+: 52.7%, T−: 22.6%). Several studies have found elevated levels of free CatB in AD blood samples,^46^ contrary to the data reported in this study (Supplementary Fig. 7A and B). The cause of this difference is presently unclear. Although further detailed studies are needed, including the roles of the extracellular efflux of CatB in association with EVs, marked changes in CatB levels in EVs may be associated with amyloid pathogenesis. These findings indicate that EV CatB might be an early biomarker for monitoring the signs of amyloid pathology and can be used for sorting subjects with AD continuum from those with normal AD biomarkers and non-AD pathologic change. EV CatB also represents an exciting new target for the development of effective therapeutic agents against AD.

As shown in Fig. 4D, the correlation between plasma EV CatB and CSF Aβ was moderate (r = −0.418), and plasma-EV-CatB levels were significantly increased in A+T−N− compared to A−T−N−, but not in the other AD continuum groups. It has been suggested that multiple brain waste efflux systems, including the glymphatic system and intramural peri-arterial drainage pathway (IPAD), are possibly impaired in AD,^47,48^ If the EV efflux pathway currently unidentified is impaired during AD progression, EV CatB efflux into blood will be reduced as the pathology progresses. Therefore, it is possible that there are significant differences in EV CatB levels between the A−T−N− and A+T−N−/A+T+N−/A+T+N+ stages in the CSF and only slight differences between A−T−N− and A+T−N− in the plasma. It is considered difficult to use EV CatB as a blood biomarker with the measurement method used in this study. Recent technological advances in microfluidics have enabled enormous progress in our ability to analyse liquid biopsy samples.^49^ Recently, digital ELISA has been used to measure target proteins at femtogram levels.^50^ Isolating brain-specific EVs might also enable more accurate detection of brain pathologies. A previous proteomic analysis and ingenuity pathway analysis (IPA) of blood EVs also identified four neuron- or oligodendrocyte-derived EV marker proteins, including syntaxin binding protein 1 (STXBP1), neuronal membrane glycoprotein M6-a (GPM6A), PH and SEC7 domain-containing protein 2 (PSD2) and Rho GDP-dissociation inhibitor 1(GDI1).^51^ Thus, a combination of these isolation technologies for brain cell-derived EVs and detection of EV-CatB could allow more sensitive and specific monitoring of AD pathogenesis in the future.

Finally, a limitation of this study is that the ATN classification itself might change depending on how the cut-off value for the ATN classification is set: (i) the ATN classification cut-off values are based on the ATN framing set, which may bias the profiling of the discovery and validation tests; (ii) recently, it was reported that neurofilament light chain (NfL) is likely to be a more appropriate indicator of neurodegeneration than tTau^52^; (iii) using a different N marker for ATN classification may change the evaluation of the proteins detected in this study for neurodegeneration. The discovery of EV CatB as a candidate biomarker in this study seemed to have a minor impact due to detecting a marker involved in early diagnosis involving Aβ.

In summary, our proteome-wide profiling of CSF EVs in the ATN classification identified significant changes in six proteins between amyloid positive and negative groups. Furthermore, ELISA analyses with 136 samples verified that CatB levels in EVs increase in CSF and plasma with amyloid progression, showing that CatB in EVs might be involved in AD amyloid pathogenesis. In addition to the proteins associated with amyloid pathology, our EV proteome analysis revealed proteins with unknown pathophysiological functions whose expression is altered in tau pathology/neurodegeneration, which may lead to the elucidation of the molecular pathogenesis of AD and the development of new therapeutic agents.

## Supplementary Material

awad361_Supplementary_Data

## Data Availability

The data that support the findings of this study are available from the corresponding author, upon reasonable request.
