# Supplementary material for: Extracellular vesicle proteome unveils cathepsin B connection to Alzheimer’s disease pathogenesis
Source: Brain. 2023 Dec 10;147(2):627–36. doi: 10.1093/brain/awad361 (PMC10834236; doi:10.1093/brain/awad361)
Supplement: awad361_Supplementary_Data [file awad361_supplementary_data.zip › brain-2023-00791-File009.pdf]

Supplementary materials

Extracellular Vesicle Proteome Unveils Cathepsin B Connection to Alzheimer's Disease Pathogenesis

Kohei Yuyama, Hui Sun, Risa Fujii, Isao Hemmi, Koji Ueda and Yukifusa Igeta

Supplementary Figure 1

ROC analysis for each ATN biomarker.

The ROC analysis was performed to discriminate between non-AD (normal + MCI) and AD groups. AUC values and corresponding cut-off values for AD diagnosis were determined for each biomarker.

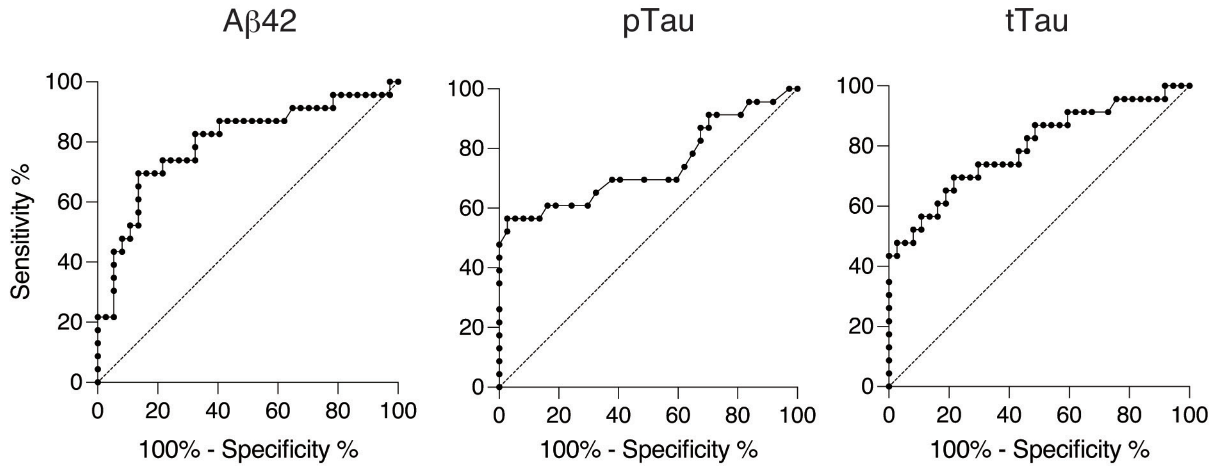

Supplementary Figure 2

Particle size distribution of the isolated EVs.

EVs were isolated from CSF by EVSecond and from CSF and plasma by ultracentrifugation method. Particle size was measured using a nanoparticle analyzer, Videodrop.

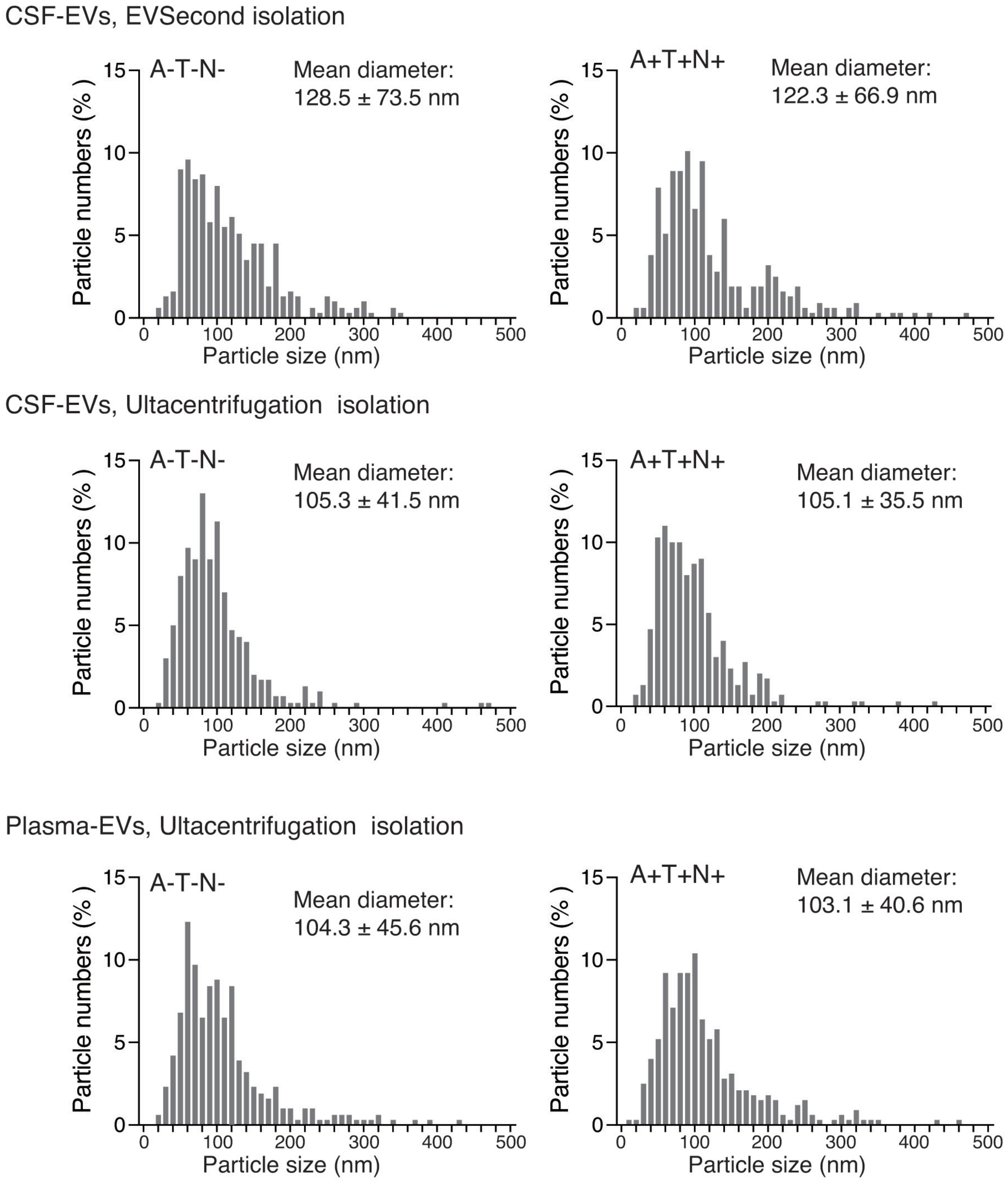

Supplementary Figure 3

Proteomic analysis of cerebrospinal fluid (CSF) extracellular vesicle (EV) proteins in discovery set under ATN classification.

(**A**) Venn diagram of the CSF EV proteins and Vesiclepedia Top100 proteins that often identified in EVs. (**B**) The gene ontology (GO) analyses for Molecular function, Biological process, and Cellular Component using DAVID Bioinformatics Resources 6.8.

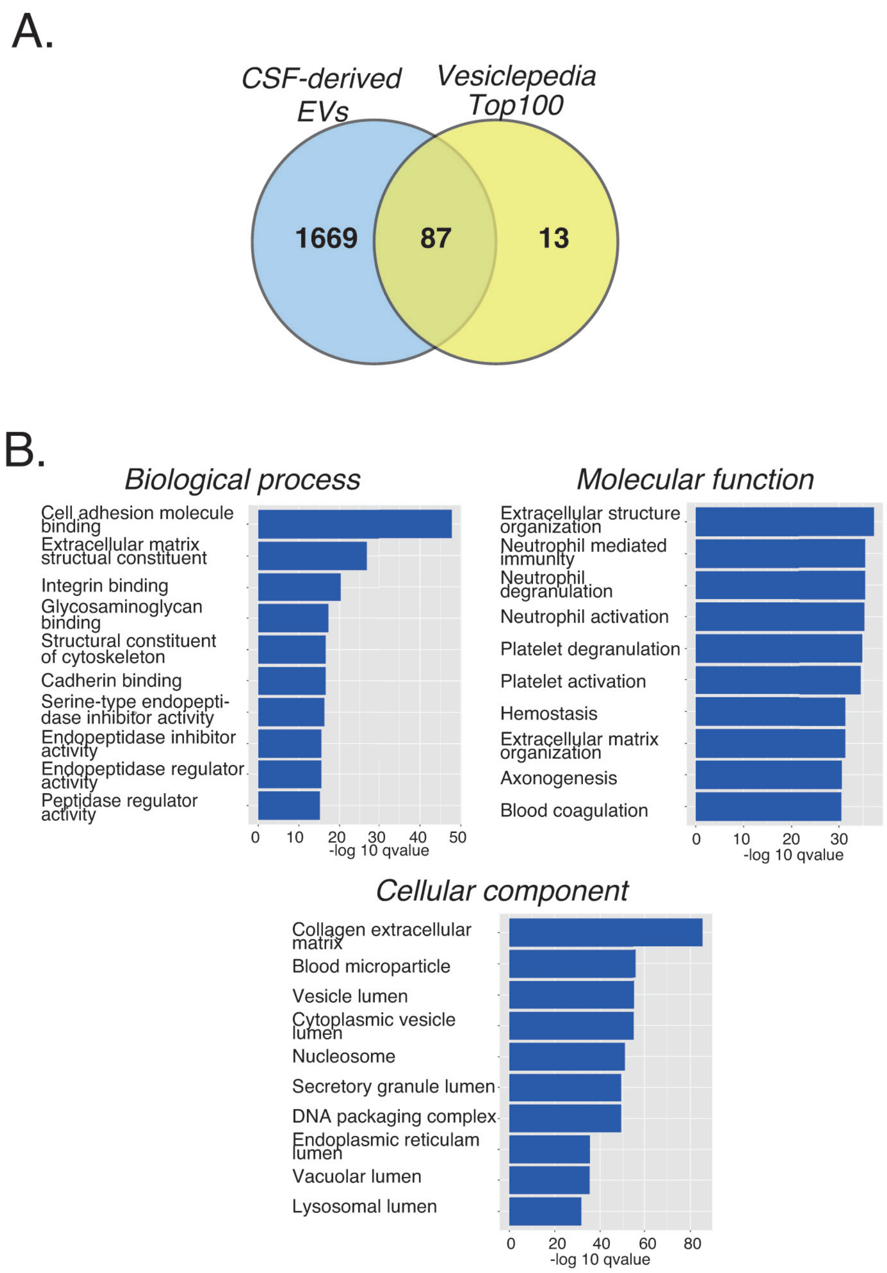

Supplementary Figure 4

The abundances of Alix, CD9, and CD63 in EVs in ATN classification, quantified by LC-MS.

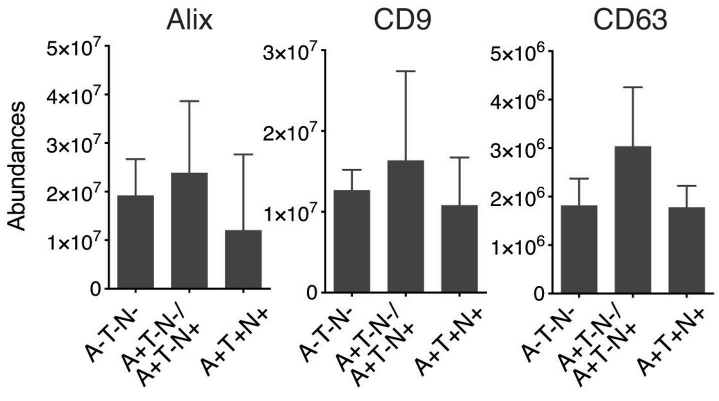

Supplementary Figure 5

Candidates for extracellular vesicle (EV) biomarkers in the ATN classification.

The abundances of EV proteins quantified by MS are shown. The EV proteins with significantly different abundances between A-T-N- and A+T-N-/A+T-N+ (A) and between A+T-N-/A+T-N+ and A+T+N+ (B) were identified as candidate biomarkers in the ATN categorization.

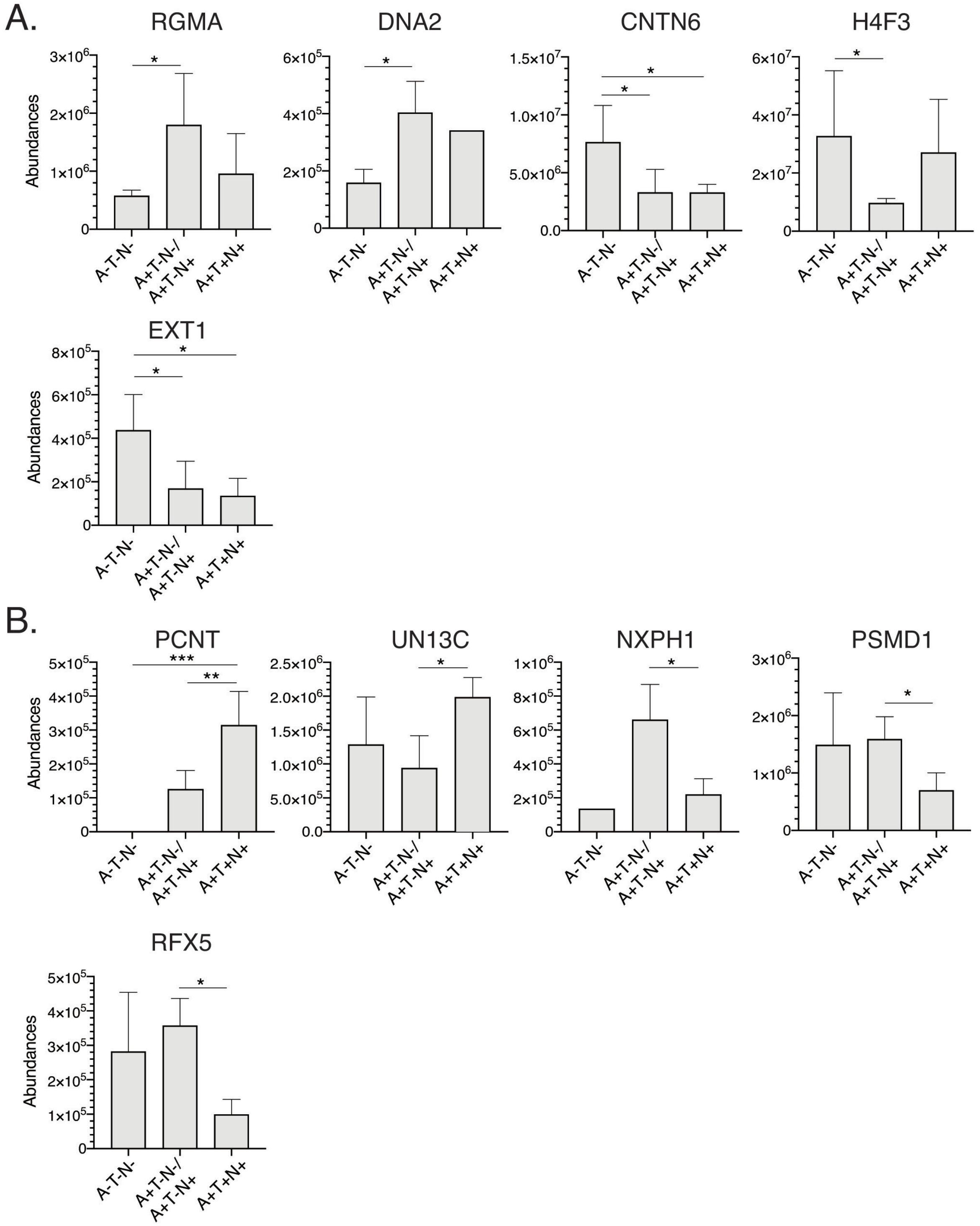

Supplementary Figure 6

The result of Sequest database search on Proteome Discoverer 2.5 software for the CATB264-281 peptide.

The criterion for peptide identification was set at FDR < 0.01.

Peptide Summary

Sequence: SGVYQHVTGEMMGGHAIR,
Charge: +4, Monoisotopic m/z: 483.23120 Da (-0.71 mmu/-1.47 ppm), MH+: 1929.90298 Da, RT: 48.9147 min,
Identified with: Sequest HT (v1.17); XCorr:2.38, Percolator q-Value:0.0e0, Percolator PEP:1.2e-5,
Fragment match tolerance used for search: 0.8 Da
Fragments used for search: y-H_2O; y-NH_3; b; b-H_2O; b-NH_3; y

Proteins (1):
- Cathepsin B OS=Homo sapiens GN=CTSB PE=1 SV=3

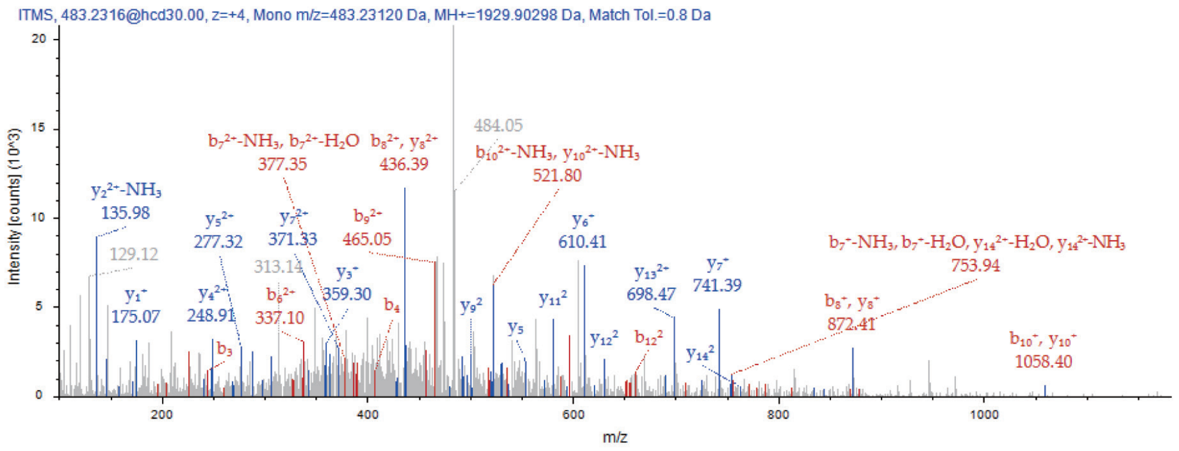

Value Type: Theo. Mass [Da]

Ion Series | Neutral Losses | Precursor Ions | Internal Fragments

#1	b+	b2+	Seq.	y+	y2+	#2
1	88.03930	44.52329	S			18
2	145.06077	73.03402	G	1842.87378	921.94053	17
3	244.12918	122.56823	V	1785.85232	893.42980	16
4	407.19251	204.09989	Y	1686.78390	843.89559	15
5	535.25109	268.12918	Q	1523.72058	762.36393	14
6	672.31000	336.65864	H	1395.66200	698.33464	13
7	771.37841	386.19285	V	1258.60309	629.80518	12
8	872.42609	436.71668	T	1159.53467	580.27098	11
9	929.44756	465.22742	G	1058.48699	529.74714	10
10	1058.49015	529.74871	E	1001.46553	501.23640	9
11	1189.53063	595.26896	M	872.42294	436.71511	8
12	1320.57112	660.78920	M	741.38245	371.19487	7
13	1377.59258	689.29993	G	610.34197	305.67462	6
14	1434.61405	717.81066	G	553.32051	277.16389	5
15	1571.67296	786.34012	H	496.29904	248.65316	4
16	1642.71007	821.85867	A	359.24013	180.12370	3
17	1755.79414	878.40071	I	288.20302	144.60515	2
18			R	175.11895	88.06311	1

Supplementary Figure 7

ELISA analysis of cathepsin B (CatB) in plasma in the ATN classification.

(A) Box plots of CatB abundances in plasma in each ATN group, quantified by ELISA. (B) Scatter plots of CatB in plasma in A+/A-, T+/ T-, and N+/N- classifications. One-way ANOVA, followed by Tukey's multiple comparisons test.

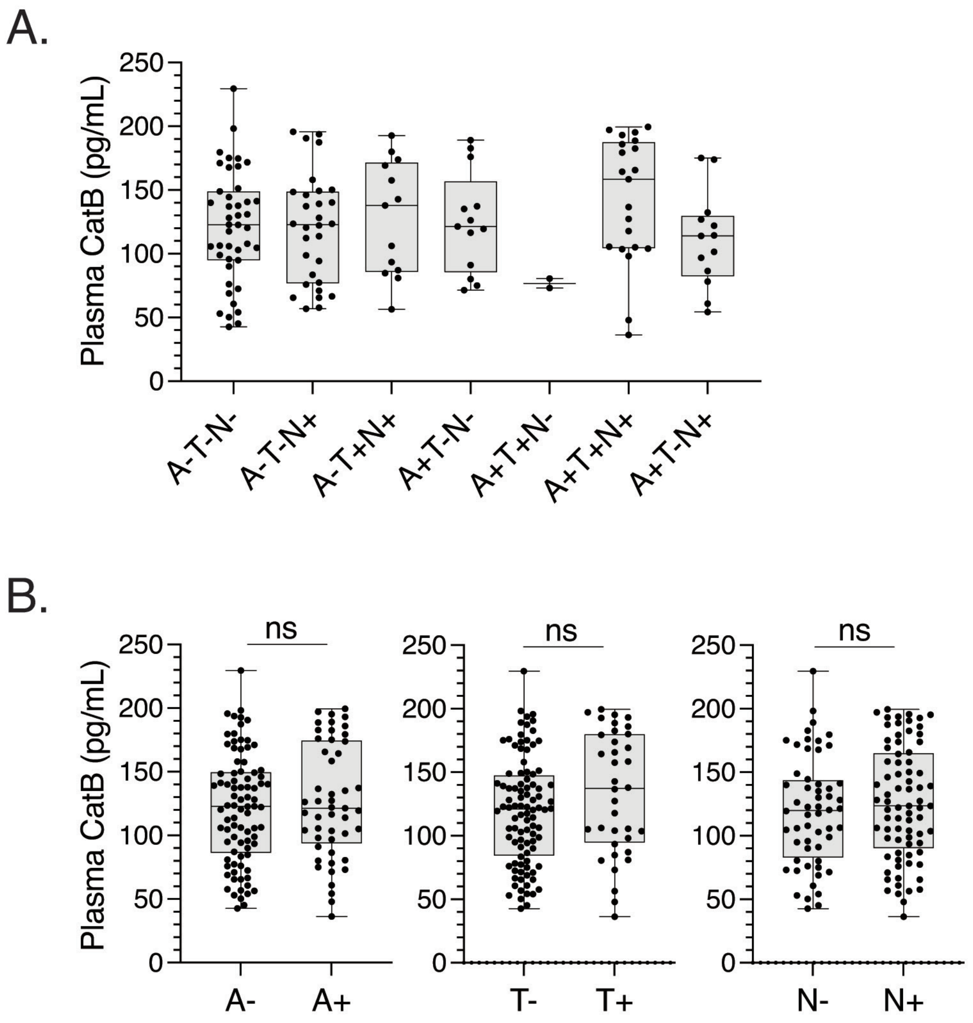

Supplementary Table 1

Patient information.

Supplementary Table 2

ROC analysis for each ATN biomarker.

Supplementary Table 3

List of CSF EV proteins.

Supplementary Table 4

EV proteins unique to A-T-N-, A+T-N-/A+T-N+, and A+T+N+.

Supplementary Table 5

EV proteins shared by the two ATN groups.

Supplementary Table 6

Up and downregulated EV proteins between A-T-N- and A+T+N+ stage transitions.
